# Is CO_2_ an Indoor Pollutant? Direct Effects of Low-to-Moderate CO_2_ Concentrations on Human Decision-Making Performance

**DOI:** 10.1289/ehp.1104789

**Published:** 2012-09-20

**Authors:** Usha Satish, Mark J. Mendell, Krishnamurthy Shekhar, Toshifumi Hotchi, Douglas Sullivan, Siegfried Streufert, William J. Fisk

**Affiliations:** 1 Department of Psychiatry and Behavioral Science, Upstate Medical University, State University of New York, Syracuse, New York, USA; 2 Indoor Environment Department, Lawrence Berkeley National Laboratory, Berkeley, California, USA

**Keywords:** carbon dioxide, cognition, decision making, human performance, indoor environmental quality, ventilation

## Abstract

Background: Associations of higher indoor carbon dioxide (CO_2_) concentrations with impaired work performance, increased health symptoms, and poorer perceived air quality have been attributed to correlation of indoor CO_2_ with concentrations of other indoor air pollutants that are also influenced by rates of outdoor-air ventilation.

Objectives: We assessed direct effects of increased CO_2_, within the range of indoor concentrations, on decision making.

Methods: Twenty-two participants were exposed to CO_2_ at 600, 1,000, and 2,500 ppm in an office-like chamber, in six groups. Each group was exposed to these conditions in three 2.5-hr sessions, all on 1 day, with exposure order balanced across groups. At 600 ppm, CO_2_ came from outdoor air and participants’ respiration. Higher concentrations were achieved by injecting ultrapure CO_2_. Ventilation rate and temperature were constant. Under each condition, participants completed a computer-based test of decision-making performance as well as questionnaires on health symptoms and perceived air quality. Participants and the person administering the decision-making test were blinded to CO_2_ level. Data were analyzed with analysis of variance models.

Results: Relative to 600 ppm, at 1,000 ppm CO_2_, moderate and statistically significant decrements occurred in six of nine scales of decision-making performance. At 2,500 ppm, large and statistically significant reductions occurred in seven scales of decision-making performance (raw score ratios, 0.06–0.56), but performance on the focused activity scale increased.

Conclusions: Direct adverse effects of CO_2_ on human performance may be economically important and may limit energy-saving reductions in outdoor air ventilation per person in buildings. Confirmation of these findings is needed.

Because humans produce and exhale carbon dioxide (CO_2_), concentrations of CO_2_ in occupied indoor spaces are higher than concentrations outdoors. As the ventilation rate (i.e., rate of outdoor air supply to the indoors) per person decreases, the magnitude of the indoor–outdoor difference in CO_2_ concentration increases. Consequently, peak indoor CO_2_ concentrations, or the peak elevations of the indoor concentrations above those in outdoor air, have often been used as rough indicators for outdoor-air ventilation rate per occupant (Persily and Dols 1990). The need to reduce energy consumption provides an incentive for low rates of ventilation, leading to higher indoor CO_2_ concentrations.

Although typical outdoor CO_2_ concentrations are approximately 380 ppm, outdoor levels in urban areas as high as 500 ppm have been reported (Persily 1997). Concentrations of CO_2_ inside buildings range from outdoor levels up to several thousand parts per million (Persily and Gorfain 2008). Prior research has documented direct health effects of CO_2_ on humans, but only at concentrations much higher than those found in normal indoor settings. CO_2_ concentrations > 20,000 ppm cause deepened breathing; 40,000 ppm increases respiration markedly; 100,000 ppm causes visual disturbances and tremors and has been associated with loss of consciousness; and 250,000 ppm CO_2_ (a 25% concentration) can cause death (Lipsett et al. 1994). Maximum recommended occupational exposure limits for an 8-hr workday are 5,000 ppm as a time-weighted average, for the Occupational Safety and Health Administration (OSHA 2012) and the American Conference of Government Industrial Hygienists (ACGIH 2011).

Epidemiologic and intervention research has shown that higher levels of CO_2_ within the range found in normal indoor settings are associated with perceptions of poor air quality, increased prevalence of acute health symptoms (e.g., headache, mucosal irritation), slower work performance, and increased absence (Erdmann and Apte 2004; Federspiel et al. 2004; Milton et al. 2000; Seppanen et al. 1999; Shendell et al. 2004; Wargocki et al. 2000). It is widely believed that these associations exist only because the higher indoor CO_2_ concentrations at lower outdoor air ventilation rates are correlated with higher levels of other indoor-generated pollutants that directly cause the adverse effects (Mudarri 1997; Persily 1997). Thus CO_2_ in the range of concentrations found in buildings (i.e., up to 5,000 ppm) has been assumed to have no direct impacts on occupants’ perceptions, health, or work performance.

Researchers in Hungary have questioned this assumption (Kajtar et al. 2003, 2006). The authors reported that controlled human exposures to CO_2_ between 2,000 ppm and 5,000 ppm, with ventilation rates unchanged, had subtle adverse impacts on proofreading of text in some trials, but the brief reports in conference proceedings provided limited details.

This stimulated our group to test effects of variation in CO_2_ alone, in a controlled environment, on potentially more sensitive high-level cognitive functioning. We investigated a hypothesis that higher concentrations of CO_2_, within the range found in buildings and without changes in ventilation rate, have detrimental effects on occupants’ decision-making performance.

## Methods

This study addresses responses among human participants under three different conditions in a controlled environmental chamber outfitted like an office, with CO_2_ concentrations of approximately 600, 1,000, and 2,500 ppm. Six groups of four participants were scheduled for exposure to each of the three conditions for 2.5 hr per condition. The experimental sessions for each group took place on a single day, at 0900–1130, 1230–1500, and 1600–1830 hours, with 1-hr breaks outside the exposure chamber between sessions. During the first break, participants ate a self-provided lunch. The order in which participants were exposed to the different CO_2_ concentrations was balanced across groups, including all possible orders of low-, medium-, and high-concentration sessions. Participants and the person administering the tests of decision-making performance were not informed about specific CO_2_ conditions in each session. During each exposure condition, participants completed a computer-based test of decision-making performance in which they were presented with scenarios and asked to make decisions based on a standardized protocol (Krishnamurthy et al. 2009; Satish et al. 2009; Streufert and Satish 1997). Before and after each test of decision-making performance, participants also completed computer-based questionnaires on perceived indoor air quality and health symptoms.

We received approval for the study protocol and the informed consent procedures from the Human Subjects Committee at Lawrence Berkeley National Laboratory (LBNL). We recruited primarily from among a local population of university students, all at least 18 years old. We scheduled 24 participants, with extras in case of no-shows, for participation. All participants provided written informed consent before participation. Scheduled participants were provided a small amount of financial compensation for their time.

*Exposure protocol.* Experimental sessions were conducted in a chamber facility at LBNL. The chamber has a 4.6 m × 4.6 m floor plan, 2.4 m high ceiling, standard gypsum board walls, and vinyl flooring, and is equipped with four small desks, each with an Internet-connected computer. The chamber is located inside a heated and cooled building, with all external surfaces of the chamber surrounded by room-temperature air. The chamber has one window (~ 1 m × 1 m) that views the interior of the surrounding indoor space; hence, changes in daylight or the view to outdoors were not factors in the research. The chamber has a relatively airtight envelope, including a door with a refrigerator-style seal. The chamber was positively pressurized relative to the surrounding space. A small heating, ventilating, and air-conditioning system served the chamber with thermally conditioned air filtered with an efficient particle filter. The outdoor air supply rate was maintained constant at approximately 3.5 times the 7.1 L/sec per person minimum requirement in California (California Energy Commission 2008); the flow rate was monitored continuously with a venturi flow meter (model VWF 555 - 4”; Gerand Engineering Co, Minneapolis, MN).

CO_2_ was recorded in real time at 1-min intervals. During the baseline sessions, with participants and outdoor air as the only indoor source of CO_2_, measured CO_2_ concentrations were approximately 600 ppm. In sessions with CO_2_ added, CO_2_ from a cylinder of ultra-pure CO_2_ (at least 99.9999% pure) was added to the chamber supply air, upstream of the supply-air fan to assure mixing of the CO_2_ in the air, at the rate needed to increase the CO_2_ concentration to either 1,000 or 2,500 ppm. A mass flow controller monitored and regulated injection rates in real time. All other conditions (e.g., ventilation rate, temperature) remained unchanged.

The outdoor air exchange rate of the chamber was about 7/hr; and in sessions with CO_2_ injected into the chamber, injection started before the participants entered the chamber. In sessions with no CO_2_ injection, CO_2_ concentrations were close to equilibrium levels 25 min after the start of occupancy, and in sessions with CO_2_ injection (because CO_2_ injection started before participants entered the chamber), 10–15 min after the start of occupancy.

Before participants entered the chamber, the desired chamber temperature and ventilation rate were established at target values of 23^o^C (73^o^F) and 100 L/sec (210 ft^3^/min). Indoor chamber temperature during the experimental sessions was maintained at approximately 23^o^C (73.4^o^F) by proportionally controlled electric resistance heating in the supply airstream. Relative humidity (RH) was approximately 50% ± 15%. We continuously monitored temperature and RH in real time. Temperature was averaged for each session for comparisons.

Calibrations of all instruments were checked at the start of the study. Calibration of the CO_2_ monitors was checked at least every week during experiments using primary standard calibration gases. Given the instruments used and calibration procedures, we anticipated measurement accuracies of ± 5% at the lowest CO_2_ concentrations and as high as ± 3% at the highest concentrations. Real-time logged environmental data (CO_2_, temperature, RH, outdoor air supply rate) were downloaded from environmental monitors to Excel and imported into SAS statistical analysis software (version 9.1; SAS Institute Inc., Cary, NC).

The design of the CO_2_ injection system included features to prevent unsafe CO_2_ concentrations from developing in the event of a failure in the CO_2_ injection system or human error. The CO_2_ cylinder was outdoors so that any leaks would be to outdoors. A pressure relief valve located downstream of the pressure regulator was also located outdoors and set to prevent pressures from exceeding our target pressure at the inlet of the mass flow controller by > 50%. Valves would automatically stop CO_2_ injection if the outdoor air ventilation to the chamber or the ventilation fan failed. A flow limiter prevented CO_2_ concentrations from exceeding 5,000 ppm if the mass flow controller failed in the fully open position, and a second CO_2_ analyzer with control system would automatically stop CO_2_ injection if the concentration exceeded 5,000 ppm. Also, a research associate monitored CO_2_ concentrations in the chamber using a real-time instrument. Given the purity level of the carbon dioxide in the gas cylinder (99.9999%) and the rate of outdoor air supply to the chamber, the maximum possible chamber air concentration of impurities originating from the cylinder of CO_2_ was only 2 ppb. The impurity of highest concentration was likely to be water vapor, and at a concentration ≤ 2 ppb, short-term health risks from exposures to impurities would have been far less than risks associated with exposures to many normal indoor or outdoor pollutants. Finally, before participants entered the chamber we added CO_2_ from the cylinder to the chamber air, and collected an air sample on a sorbent tube for analysis by thermal desorption gas chromatography mass spectrometry. There was no evidence that the CO_2_ injection process increased indoor concentrations of volatile organic compounds (VOCs). VOCs at low concentrations, typical of indoor and outdoor air concentrations, were detected.

On the morning of each of 6 experimental days, groups of participants came to LBNL for a full day of three experimental sessions. To ensure a full set of four participants for each scheduled day (after one unanticipated no-show on each of the first 2 days), we scheduled five participants each day and selected four at random to participate. On each experimental day, as soon as all participants had arrived, the selected participants were seated in the environmental chamber facility. Before they entered the chamber, a research associate distributed to participants a handout describing the session plans and answered any questions.

During the first 45 min of each session, participants were free to perform school work, read, or engage in any quiet, nondisruptive activity. Participants were then asked by the LBNL research associate to complete the computer-based questionnaire on perceived air quality and symptoms, available via web connection on the laptop computers on their desks. Participants then had a 10-min break, to stretch or exit the chamber to use the bathroom, but no participant elected to exit the chamber during a session.

A 20-min protocol was then used to train participants in the decision-making task. A technician trained in administering this test was present to answer questions before the test, and could enter the chamber to answer questions during the test. We estimated that CO_2_ emissions of the technician, who was in the chamber for about 10 min during each session, would increase chamber CO_2_ concentrations by no more than 17 ppm. (The technician was not required to give informed consent for this because the study conditions are commonly experienced in indoor environments and are not associated with adverse health effects.) Over the next 1.5 hr, participants took the computerized test of decision-making performance, which involved reading text displayed on a laptop computer and selecting among possible responses to indicate their decisions.

When the performance test was completed, participants repeated the computer-based questionnaire on perceived air quality and symptoms and then left the chamber until the next session. At any time during each session, participants were free to exit the facility to use a nearby bathroom, but were asked to return within 10 min. Participants were also free to terminate their participation and leave the facility at any time during the day, but no participants exercised these options.

*Testing of decision-making performance.* We used a testing method designed to assess complex cognitive functioning in ways more relevant to the tasks of workers in buildings than the tests of simulated office work generally used (e.g., proofreading text, adding numbers) (Wargocki et al. 2000). A computer-based program called the Strategic Management Simulation (SMS) test collects data on performance in decision making under different conditions. The SMS test has been used to study the impact on people’s decision-making abilities of different drugs, VOCs from house painting, stress overload, head trauma, and the like (Breuer and Satish 2003; Cleckner 2006; Satish et al. 2004, 2008; Swezey et al. 1998). (SMS testing is available for research by contract with State University of New York Upstate Medical University, and for commercial applications via Streufert Consulting, LLC. See http://www.upstate.edu/psych/research/sms.php.)

The SMS measures complex human behaviors required for effectiveness in many workplace settings. The system assesses both basic cognitive and behavioral responses to task demands, as well as cognitive and behavioral components commonly considered executive functions. The system and its performance have been described in prior publications (e.g., Breuer and Satish 2003; Satish et al. 2004; Swezey et al. 1998). Participants are exposed to diverse computer-generated situations presenting real-world equivalent simulation scenarios that are proven to match real-world day-to-day challenges. Several parallel scenarios are available, allowing retesting individuals without bias due to experience and learning effects. Participants are given instructions via text messages on a user-friendly computer interface, and respond to the messages using a drop-down menu of possible decisions. All participants receive the same quantity of information at fixed time points in simulated time, but participants have flexibility to take actions and make decisions at any time during the simulation, as in the real world. The absence of requirements to engage in specific actions or to make decisions at specific points in time, the absence of stated demands to respond to specific information, the freedom to develop initiative, and the freedom for strategy development and decision implementation allow each participant to use his or her own preferred or typical action, planning, and strategic style. The SMS system generates measurement profiles that reflect the underlying decision-making capacities of the individual.

The computer calculates SMS performance measures as raw scores, based on the actions taken by the participants, their stated future plans, their responses to incoming information, and their use of prior actions and outcomes. The validated measures of task performance vary from relatively simple competencies such as speed of response, activity, and task orientation, through intermediate level capabilities such as initiative, emergency responsiveness, and use of information, to highly complex thought and action processes such as breadth of approach to problems, planning capacity, and strategy. The nine primary factors and factor combinations that have predicted real-world success are basic activity level (number of actions taken), applied activity (opportunistic actions), focused activity (strategic actions in a narrow endeavor), task orientation (focus on concurrent task demands), initiative (development of new/creative activities), information search (openness to and search for information), information usage (ability to use information effectively), breadth of approach (flexibility in approach to the task), and basic strategy (number of strategic actions).

The raw scores assigned for each measure are linearly related to performance, with a higher score indicating superior performance. Interpretation is based on the relationship to established standards of performance excellence among thousands of previous SMS participants (Breuer and Streufert 1995; Satish et al. 2004, 2008; Streufert and Streufert 1978; Streufert et al. 1988; Streufert and Swezey 1986). Percentile ranks are calculated through a comparison of raw scores to the overall distribution of raw scores from a reference population of > 20,000 U.S. adults, 16–83 years of age, who had previously completed the SMS. The reference population was constructed nonrandomly to be generally representative of the job distribution among the adult U.S. population, including, for example, college students, teachers, pilots, medical residents, corporate executives, homemakers, and the unemployed. The percentile calculations for individual participants are not further adjusted for age, sex, or education level.

*Data management and analysis.* The main predictor variable of interest was CO_2_, included in analyses as a categorical variable with three values: 600, 1,000, and 2,500 ppm. Real-time CO_2_ concentrations and temperature were averaged for each session for comparison.

Nine measures from the SMS, representing validated independent assessments of performance in complex task settings, were compared across CO_2_ conditions. Raw scores on the different SMS measures were computer-calculated based on procedures (software formulas) that are discussed by Streufert and Swezey (1986). The formulas are based on numerically and graphically scored decision actions, on the interrelationships among decisions over time, the interrelationships among decisions with incoming information, as well as decision planning and other components of participant activity. Each of the activity event components that are used in the formulas are collected by the SMS computer software program (Streufert and Swezey 1986). A separate SMS software system is subsequently used to calculate the value for each measure. Where appropriate—where maximum performance levels have limits (cannot be exceeded)—the obtained scores are expressed by the program as percentages of maximally obtainable values. A higher score on a measure indicates better performance in that area of performance. For each measure, ratios of scores across conditions were calculated to show the magnitude of changes.

Initial data analysis used multivariate analysis of variance (MANOVA) to assess overall significance across all conditions, to assure that subsequent (post hoc) analysis across the nine different simulation measures would be legitimate. With high levels of significance established, post hoc analysis for each simulation measure using analysis of variance (ANOVA) techniques becomes possible. Separate ANOVA procedures across CO_2_ conditions were used for each of the nine SMS measures (within participants, with participants as their own controls). Percentile ranks were calculated from the raw scores and normative data, without adjustments for demographic or other variables. Percentile levels are divided into categories with descriptive labels based on prior test findings from different populations, normal and impaired.

## Results

Because 2 of the 24 originally scheduled participants cancelled at a time when they could not be replaced, 22 participants provided complete SMS data. Of these, 10 were male; 18 were 18–29 years of age, and 4 were 30–39 years of age. One participant had completed high school only, 8 had completed some college, and 13 had a college degree. None were current smokers, 1 reported current asthma, and 5 reported eczema, hay fever, or allergy to dust or mold.

Median CO_2_ values for the low, medium, and high CO_2_ conditions were 600, 1,006, and 2,496 ppm (which we refer to as 600, 1,000, and 2,500 ppm), and ranges were 132, 92, and 125 ppm, respectively (Table 1). Temperatures in the study chamber were controlled effectively, varying overall within about 0.2^o^C (from 22.9 to 23.1^o^C in each condition), and with median values across the three CO_2_ conditions varying < 0.1^o^C.

**Table 1:** CO_2_ concentrations during study conditions.

CO2 condition	CO2 concentration (ppm)						
	Minimum	Median	Maximum	Range
Low	542	600	675	132
Medium	969	1,006	1,061	92
High	2,418	2,496	2,543	125
Overall	542	1,006	2,543	—

**Figure 1:**
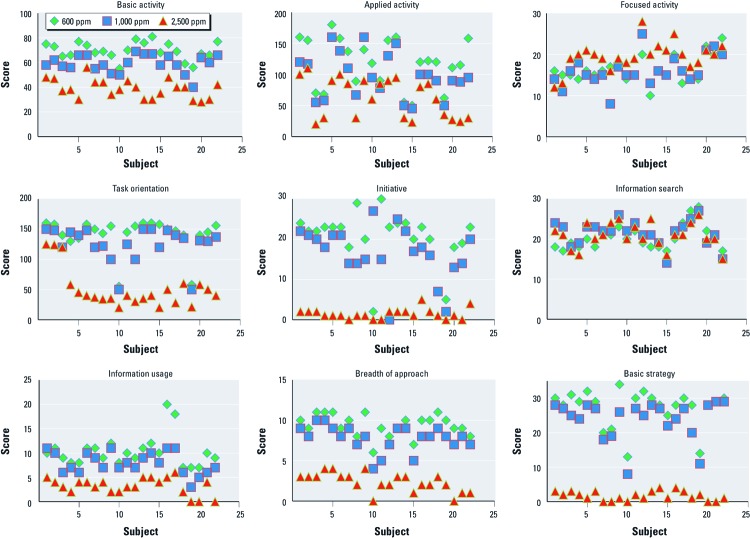
Plots of individual scores, by condition, for each of the SMS measures of decision-making performance (*n* = 22 subjects).

The raw scores for each of the SMS performance measures were plotted for each participant according to CO_2_ level (Figure 1). The plots indicate clear relationships between raw scores and CO_2_ level for all performance measures other than focused activity and information search, with dramatic reductions in raw scores at 2,500 ppm CO_2_ for some measures of decision-making performance.

For seven of nine scales of decision-making performance (basic activity, applied activity, task orientation, initiative, information usage, breadth of approach, and basic strategy), mean raw scores showed a consistently monotonic decrease with increasing CO_2_ concentrations, with all overall *p*-values < 0.001 (Table 2). In post hoc pairwise comparisons by CO_2_ concentration, performance on these seven scales differed between concentrations with *p* < 0.01 for all comparisons, except for performance on the task orientation, initiative, and basic strategy scales between 600 and 1,000 ppm CO_2_ (*p* < 0.05, *p* < 0.10, and *p* < 0.05, respectively) (Table 3). For these seven scales, compared with mean raw scores at 600 ppm CO_2_, mean raw scores at 1,000 ppm CO_2_ were 11–23% lower, and at 2,500 ppm CO_2_ were 44–94% lower. Relative to raw scores at 1,000 ppm CO_2_, raw scores at 2,500 ppm were 35–93% lower.

**Table 2:** Mean raw scores for nine outcome variables at three conditions of CO_2_ concentration among 22 participants, and comparison using MANOVA.

Outcome variables	Conditions (ppm of CO2) (mean ± SD)			Overall F-statistic (df = 2,42)	p-Value
	600 ppm	1,000 ppm	2,500 ppm		
Basic activity	69.59 ± 7.04	59.23 ± 7.12	38.77 ± 7.57	172.77	< 0.001
Applied activity	117.86 ± 39.28	97.55 ± 35.51	62.68 ± 31.86	72.13	< 0.001
Focused activity	16.27 ± 3.20	16.09 ± 3.70	19.55 ± 3.40	17.26	< 0.001
Task orientation	140.82 ± 28.66	125.41 ± 28.62	50.45 ± 31.66	115.08	< 0.001
Initiative	20.09 ± 6.96	16.45 ± 6.70	1.41 ± 1.26	81.45	< 0.001
Information search	20.36 ± 3.06	21.5 ± 3.20	20.91 ± 3.08	2.51	> 0.10
Information usage	10.32 ± 3.21	7.95 ± 2.24	3.18 ± 1.71	129.20	< 0.001
Breadth of approach	9.36 ± 1.36	7.82 ± 1.56	2.32 ± 1.17	679.88	< 0.001
Basic strategy	27.23 ± 5.48	23.95 ± 5.65	1.68 ± 1.32	414.51	< 0.001
df, degrees of freedom.										

**Table 3:** Comparison of mean raw scores for nine decision-making measures between three different CO_2_ concentrations among 22 participants.

Variables	Ratios of condition scoresa				
	Score at 1,000 ppm/score at 600 ppm	Score at 2,500 ppm/score at 1,000 ppm	Score at 2,500 ppm/score at 600 ppm
Basic activity	0.85#	0.65#	0.56#
Applied activity	0.83#	0.64#	0.53#
Focused activity	0.99	1.22#	1.20#
Task orientation	0.89**	0.40#	0.36#
Initiative	0.82*	0.09#	0.07#
Information search	1.06	0.97	1.03
Information usage	0.77#	0.40#	0.31#
Breadth of approach	0.84#	0.30#	0.25#
Basic strategy	0.88**	0.07#	0.06#
df, degrees of freedom. ap-Values based on F-test, df = 1,21, calculated for difference between score in numerator and score in denominator. *p < 0.10. **p < 0.05. #p < 0.01.						

For information search, mean raw scores were similar at all three CO_2_ conditions. Neither the overall analysis across the three conditions (Table 2) nor the post hoc pairwise analyses (Table 3) indicated significant differences. For focused activity, raw scores at 600 ppm CO_2_ and 1,000 ppm CO_2_ were nearly identical (16.27 and 16.09), but the mean raw score at 2,500 ppm was higher (19.55), resulting in an overall *p*-value ≤ 0.001 (Table 2). Post hoc tests indicated no difference between mean raw scores at 600 and 1,000 ppm CO_2_, but significant differences (*p* ≤ 0.01) between the mean raw score at 2,500 ppm CO_2_ and scores at both 600 and 1,000 ppm (Table 3).

**Figure 2:**
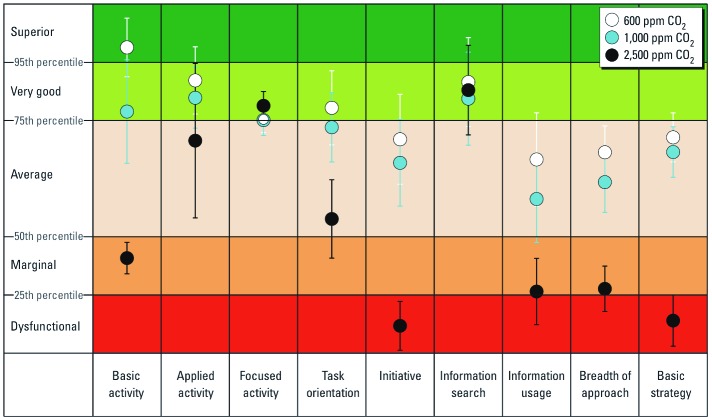
Impact of CO_2_ on human decision-making performance. Error bars indicate 1 SD.

Figure 2 shows the percentile scores on the nine scales at the three CO_2_ conditions (based on the raw scores shown in Table 2), with the percentile boundaries for five normative levels of performance: superior, very good, average, marginal, and dysfunctional. At 1,000 ppm CO_2_ relative to 600 ppm, percentile ranks were moderately diminished at most. However, at 2,500 ppm CO_2_, percentile ranks for five performance scales decreased to levels associated with marginal or dysfunctional performance.

## Discussion

*Synthesis and interpretation of findings.* Performance for six of nine decision-making measures decreased moderately but significantly at 1,000 ppm relative to the baseline of 600 ppm, and seven decreased substantially at 2,500 ppm. For an eighth scale, “information search,” no significant differences were seen across conditions. In contrast to other scales, an inverse pattern was seen for “focused activity,” with the highest level of focus obtained at 2,500 ppm and the lowest at 600 ppm.

Thus, most decision-making variables showed a decline with higher concentrations of CO_2_, but measures of focused activity improved. Focused activity is important for overall productivity, but high levels of focus under nonemergency conditions may indicate “overconcentration.” Prior research with the SMS has shown repeatedly that individuals who experience difficulty in functioning [e.g., persons with mild-to-moderate head injuries (Satish et al. 2008), persons under the influence of alcohol (Streufert et al. 1993), and persons suffering from allergic rhinitis (Satish et al. 2004)] tend to become highly focused on smaller details at the expense of the big picture.

High levels of predictive validity for the SMS (*r* > 0.60 with real-world success as judged by peers and as demonstrated by income, job level, promotions, and level in organizations), as well as high levels of test–retest reliability across the four simulation scenarios (*r* = 0.72–0.94) have repeatedly been demonstrated (Breuer and Streufert 1995; Streufert et al. 1988). Additional validity is demonstrated by the deterioration of various performance indicators with 0.05% blood alcohol intoxication and seriously diminished functioning with intoxication at the 0.10 level (Satish and Streufert 2002). Baseline scores at 600 ppm CO_2_ for the participants in this study, mostly current science and engineering students from a top U.S. university, were all average or above.

Although the modest reductions in multiple aspects of decision making seen at 1,000 ppm may not be critical to individuals, at a societal level or for employers an exposure that reduces performance even slightly could be economically significant. The substantial reductions in decision-making performance with 2.5-hr exposures to 2,500 ppm CO_2_ indicate, per the available norms for the SMS test, impairment that is of importance even for individuals. These findings provide initial evidence for considering CO_2_ as an indoor pollutant, not just a proxy for other pollutants that directly affect people.

*CO_2_ concentrations in practice.* The real-world significance of our findings, if confirmed, would depend on the extent to which CO_2_ concentrations are ≥ 1,000 and ≥ 2,500 ppm in current or future buildings. There is strong evidence that in schools, CO_2_ concentrations are frequently near or above the levels associated in this study with significant reductions in decision-making performance. In surveys of elementary school classrooms in California and Texas, average CO_2_ concentrations were > 1,000 ppm, a substantial proportion exceeded 2,000 ppm, and in 21% of Texas classrooms peak CO_2_ concentration exceeded 3,000 ppm (Corsi et al. 2002; Whitmore et al. 2003). Given these concentrations, we must consider the possibility that some students in high-CO_2_ classrooms are disadvantaged in learning or test taking. We do not know whether exposures that cause decrements in decision making in the SMS test will inhibit learning by students; however, we cannot rule out impacts on learning. We were not able to identify CO_2_ measurements for spaces in which students take tests related to admission to universities or graduate schools, or from tests related to professional accreditations, but these testing environments often have a high occupant density, and thus might have elevated CO_2_ levels.

In general office spaces within the United States, CO_2_ concentrations tend to be much lower than in schools. In a representative survey of 100 U.S. offices (Persily and Gorfain 2008), only 5% of the measured peak indoor CO_2_ concentrations exceeded 1,000 ppm, assuming an outdoor concentration of 400 ppm. One very small study suggests that meeting rooms in offices, where important decisions are sometimes made, can have elevated CO_2_ concentrations—for example, up to 1,900 ppm during 30- to 90-min meetings (Fisk et al. 2010).

In some vehicles (aircraft, ships, submarines, cars, buses, and trucks), because of their airtight construction or high occupant density, high CO_2_ concentrations may be expected. In eight studies within commercial aircraft, mean CO_2_ concentrations in the passenger cabins were generally > 1,000 ppm and ranged as high as 1,756 ppm, and maximum concentrations were as high as 4,200 ppm (Committee on Air Quality in Passenger Cabins of Commercial Aircraft 2002). We did not identify data on CO_2_ concentrations in automobiles and trucks. One small study (Knibbs et al. 2008) reported low ventilation rates in vehicles with ventilation systems in the closed or recirculated-air positions. From those results, and using an assumption of one occupant and a 0.0052 L/sec CO_2_ emission rate per occupant (Persily and Gorfain 2008), we estimated steady-state CO_2_ concentrations in an automobile and pickup truck of 3,700 ppm and 1,250 ppm, respectively, above outdoor concentrations. These numbers would increase in proportion to the number of occupants. It is not known whether the findings of the present study apply to the decision making of vehicle drivers, although such effects are conceivable.

There is evidence that people wearing masks for respiratory protection may inhale air with highly elevated CO_2_ concentrations. In a recent study, dead-space CO_2_ concentrations within a respirator (i.e., N95 mask) were approximately 30,000 ppm (Roberge et al. 2010), suggesting potentially high CO_2_ concentration in inhaled air. The inhaled concentration would be lower than that within the mask, diluted by approximately 500 mL per breath inhaled through the mask. Although the study did not report the actual inhaled-air CO_2_ concentrations, partial pressures of CO_2_ in blood did not differ with wearing the mask. Caretti (1999) reported that respirator wear with low-level activity did not adversely alter cognitive performance or mood.

*Findings by others.* The Hungarian studies briefly reported by Kajtar et al. (2003, 2006) were the only prior studies on cognitive effects of moderate CO_2_ elevations that we identified. In these studies, the ventilation rate in an experimental chamber was kept constant at a level producing a chamber CO_2_ concentration of 600 ppm from the occupant-generated CO_2_; in some experiments, however, the chamber CO_2_ concentration was increased above 600 ppm, to as high as 5,000 ppm, by injecting 99.995% pure CO_2_ from a gas cylinder into the chamber. In two series of studies, participants blinded to CO_2_ concentrations performed proofreading significantly more poorly in some but not all sessions with CO_2_ concentrations of 4,000 ppm relative to 600 ppm. Similar, marginally significant differences were seen at 3,000 versus 600 ppm. (Differences were seen only in proportion of errors found, not in speed of reading.) The studies by Kajtar et al. (2003, 2006) were small (e.g., 10 participants) and found only a few significant associations out of many trials; these results may have been attributable to chance, but they did suggest that CO_2_ concentrations found in buildings may directly influence human performance. Our research, which was motivated by the Hungarian studies, involved lower concentrations of CO_2_, a larger study population, and different methods to assess human performance.

Prior studies on CO_2_ exposures, mostly at higher levels, have focused on physiologic effects. CO_2_ is the key regulator of respiration and arousal of behavioral states in humans (Kaye et al. 2004). The initial effects of inhaling CO_2_ at higher concentrations are increased partial pressure of CO_2_ in arterial blood (PaCO_2_) and decreased blood pH. However, PaCO_2_ is tightly regulated in healthy humans through reflex control of breathing, despite normal variation within and between individuals (Bloch-Salisbury et al. 2000). Inhaled CO_2_ at concentrations of tens of thousands of parts per million has been associated with changes in respiration, cerebral blood flow, cardiac output, and anxiety (Brian 1998; Kaye et al. 2004; Lipsett et al. 1994; Roberge et al. 2010; Woods et al. 1988). Little research has documented physiological impacts of moderately elevated CO_2_ concentrations, except one small study that reported changes in respiration, circulation, and cerebral electrical activity at 1,000 ppm CO_2_ (Goromosov 1968).

We do not have hypotheses to explain why inhaling moderately elevated CO_2_, with the expected resulting increases in respiration, heart rate, and cardiac output to stabilize PaCO_2_, would affect decision-making performance. Bloch-Salisbury et al. (2000) have summarized prior knowledge on effects of elevated PaCO_2_. PaCO_2_ has a direct linear relationship with cerebral blood flow in a broad range above and below normal levels, through dilation and constriction of arterioles. Moderately elevated (or reduced) PaCO_2_ has dramatic effects on central nervous system and cortical function. Bloch-Salisbury et al. (2000) reported that experimental changes in PaCO_2_ in humans within the normal range (in 2-hr sessions involving special procedures to hold respiration constant and thus eliminate the normal reflex control of PaCO_2_ through altered breathing), showed no effects on cognitive function or alertness but caused significant changes in electroencephalogram power spectra.

*Limitations.* This study successfully controlled the known environmental confounding factors of temperature and ventilation rate. Although exposures to CO_2_ in prior sessions may theoretically have affected performance in subsequent sessions, such carryover effects should not invalidate study results because of the balanced order of exposures. Suggestion effects were unlikely, because participants and the researcher explaining the SMS to them were blinded to specific conditions of each session. Although we conclude that the causality of the observed effects is clear, the ability to generalize from this group of college/university students to others is uncertain. Effects of CO_2_ between 600 and 1,000 ppm and between 1,000 and 2,500 ppm, and effects for longer and shorter periods of time are also uncertain. The strength of the effects seen at 2,500 ppm CO_2_ is so large for some metrics as to almost defy credibility, although it is possible that such effects occur without recognition in daily life. Replication of these study findings, including use of other measures of complex cognitive functioning and measures of physiologic response such as respiration and heart rate, is needed before definitive conclusions are drawn.

*Implications for minimum ventilation standards.* The findings of this study, if replicated, would have implications for the standards that specify minimum ventilation rates in buildings, and would also indicate the need to adhere more consistently to the existing standards. Many of the elevated CO_2_ concentrations observed in practice are a consequence of a failure to supply the amount of outdoor air specified in current standards; however, even the minimum ventilation rates in the leading professional standard [American Society of Heating, Refrigerating and Air-Conditioning Engineers (ASHRAE) 2010] correspond to CO_2_ concentrations > 1,000 ppm in densely occupied spaces. There is current interest in reducing ventilation rates and the rates required by standards, to save energy and reduce energy-related costs. Yet large reductions in ventilation rates could lead to increased CO_2_ concentrations that may adversely affect decision-making performance, even if air-cleaning systems or low-emission materials were used to control other indoor pollutants. It seems unlikely that recommended minimum ventilation rates in future standards would be low enough to cause CO_2_ levels > 2,500 ppm, a level at which decrements in decision-making performance in our findings were large, but standards with rates that result in 1,500 ppm of indoor CO_2_ are conceivable.

## Conclusions

Increases in indoor CO_2_ concentrations resulting from the injection of ultrapure CO_2_, with all other factors held constant, were associated with statistically significant and meaningful reductions in decision-making performance. At 1,000 ppm CO_2_, compared with 600 ppm, performance was significantly diminished on six of nine metrics of decision-making performance. At 2,500 ppm CO_2_, compared with 600 ppm, performance was significantly reduced in seven of nine metrics of performance, with percentile ranks for some performance metrics decreasing to levels associated with marginal or dysfunctional performance. The direct impacts of CO_2_ on performance indicated by our findings may be economically important, may disadvantage some individuals, and may limit the extent to which outdoor air supply per person can be reduced in buildings to save energy. Confirmation of these findings is needed.
